# Innate Lymphoid Cells at the Maternal-Fetal Interface in Human Pregnancy

**DOI:** 10.7150/ijbs.38264

**Published:** 2020-01-30

**Authors:** Rui-Qi Chang, Wen-Jie Zhou, Da-Jin Li, Ming-Qing Li

**Affiliations:** 1 Laboratory for Reproductive Immunology, NHC Key Lab of Reproduction Regulation (Shanghai Institute of Planned Parenthood Research), Hospital of Obstetrics and Gynecology, Fudan University Shanghai Medical College, Shanghai 200082, People's Republic of China.; 2 Shanghai Key Laboratory of Female Reproductive Endocrine Related Diseases, Shanghai, 200011, People's Republic of China.

**Keywords:** innate lymphoid cell, pregnancy, trophoblast invasion, spiral artery remodeling, reproductive failure, preeclampsia.

## Abstract

Pregnancy constitutes a major challenge to the maternal immune system, which must tolerate fetal alloantigen encoded by paternal genes. In addition to their role in inducing maternal-fetal immune tolerance, accumulating evidence indicates that decidual immune cells are involved in several processes required for a successful pregnancy, including trophoblast invasion as well as tissue and spiral artery remodeling. Innate lymphoid cells (ILCs), an important branch of the innate immune system, which has expanded rapidly in recent years, are strong actors in mucosal immunity, tissue homeostasis and metabolism regulation. With the recent identification of ILCs in the human decidua, the role of ILCs at the maternal-fetal interface raises concern. Herein, we review the presence and characterization of ILCs in the human decidua, as well as their function in normal pregnancy and pathological pregnancy, including reproductive failure, preeclampsia and others.

## Introduction

In human pregnancies, the embryo implants into the specialized mucosal (decidua) wall of the uterus. During early pregnancy, maternal-fetal immune tolerance contributes to embryo antigen specific tolerance, trophoblast invasion, spiral artery remodeling and embryonic development and is quite vital for the maintenance of normal pregnancy in mice and humans 1-4. Once this balance is broken, diseases associated with disturbances of maternal-fetal immune regulation, such as reproductive failure, preeclampsia and intrauterine growth retardation may occur 5-8. Therefore, the mechanism of maternal-fetal immune regulation is a hotspot worldwide. In human early pregnancy decidua, the main population of lymphocytes is natural killer (NK) cells (accounting for 70%), and the importance of which is self-evident 9. Innate lymphoid cells (ILCs), as part of the innate immune system, contain NK cells and have recently received attention from researchers. Considering the possibility for fundamental differences in the biology between mouse and human, the evidence mentioned in this article is based on studies in human ILCs at the maternal-fetal interface, unless otherwise specified. However, knockout mice are still powerful tools, especially in the discovery of transcription factors essential for the development and function of ILCs.

ILCs, as part of the innate immune systems, are characterized by a classic lymphoid cell morphology, dependence on the common cytokine receptor γ-chain and the transcriptional repressor inhibitor of DNA binding 2 (ID2) for their development, and lack of any antigen receptors 10-12. For decades, only NK cells (which were first discovered in the spleen of mice in 1975 13) and lymphoid tissue inducer (LTi) cells (which were first discovered in the lymph node of mice in 1997 14) were encompassed in the ILC family. However, quite recently, the ILC family has expanded dramatically and is divided into two distinct lineages, according to the developmental pathways, NK cells and non-cytotoxic helper ILCs 12, 15. The latter furthermore falls into three categories, including group 1 ILCs (ILC1s), group 2 ILCs (ILC2s) and group 3 ILCs (ILC3s) 15, 16. This categorization of non-cytotoxic helper ILCs reflects their differential requirement of transcription factors during development and their cytokine expression profiles 12.

As the only ILC subset to exhibit cytotoxicity, NK cells are defined as producing interferon (IFN)-γ and tumor necrosis factor in response to IL-12, IL-15 and IL-18, and expressing CD56 and CD94 in humans (Table 1) 16. The transcription factor T-bet and Eomesodermin (Eomes) are crucial for their differentiation and acquisition of function 17, 18. Similar to NK cells, ILC1s share the same pattern of cytokine production and the requirement of T-bet. Considering the resemblances between ILC1s and NK cells, and their inability to produce perforin and granzyme B, ILC1s are also described as non-cytotoxic NK-like cells 19, 20.

Human ILC2s are defined by the expression of CD127, CD161 and the chemo-attractant receptor-homologous molecule expressed on Th2 cells 21; their production of Th2-cell-associated cytokines (including IL-4, IL-5, IL-9, IL-13, and amphiregulin) in response to IL-25, IL-33 and thymic stromal lymphopoietin^22^-^24^; and the requirement of the transcription factor GATA-binding protein 3 (GATA3) and retinoic acid receptor-related orphan receptor-α (RORα) for their development 25, 26.

Finally, ILC3s, which contain several cell types, are characterized by their capacity to produce Th17-cell-associated cytokines (IL-17 and/or IL-22), following stimulation by IL-23 and IL-1β, and their dependence on the transcription factor retinoid-related orphan receptor γt (RORγt) during their development process. LTi cells, the prototypical ILC3 population, are vital factors in the formation of secondary lymphoid organs during embryogenesis. The remaining subsets of ILC3s are classified according to their expression of the natural cytotoxicity receptor (NCR), NKp44 in humans or NKp46 in mice. NCR^+^ILC3s, also known as NK22 cells 27, NCR22 cells 28, NCR^-^LTi cells 29 and ILC22s 30, produce IL-22 but not IL-17A, and require T-bet and aryl hydrocarbon receptor (AHR), in addition to RORγt, for their differentiation. Notably, CD56, one of the most typical markers of NK cells, is also found on some NCR^+^ILC3s, while NK cells are unable to produce IL-22 27, 31. NCR^-^ILC3s, which phenotypically mirror LTi cells, express the chemokine receptor CCR6 and are termed LTi-like ILC3s. Unlike LTi cells, AHR is also crucial for the development of NCR^-^ILC3s 32.

## The Composition of the Maternal-Fetal Interface

**Figure 1:**
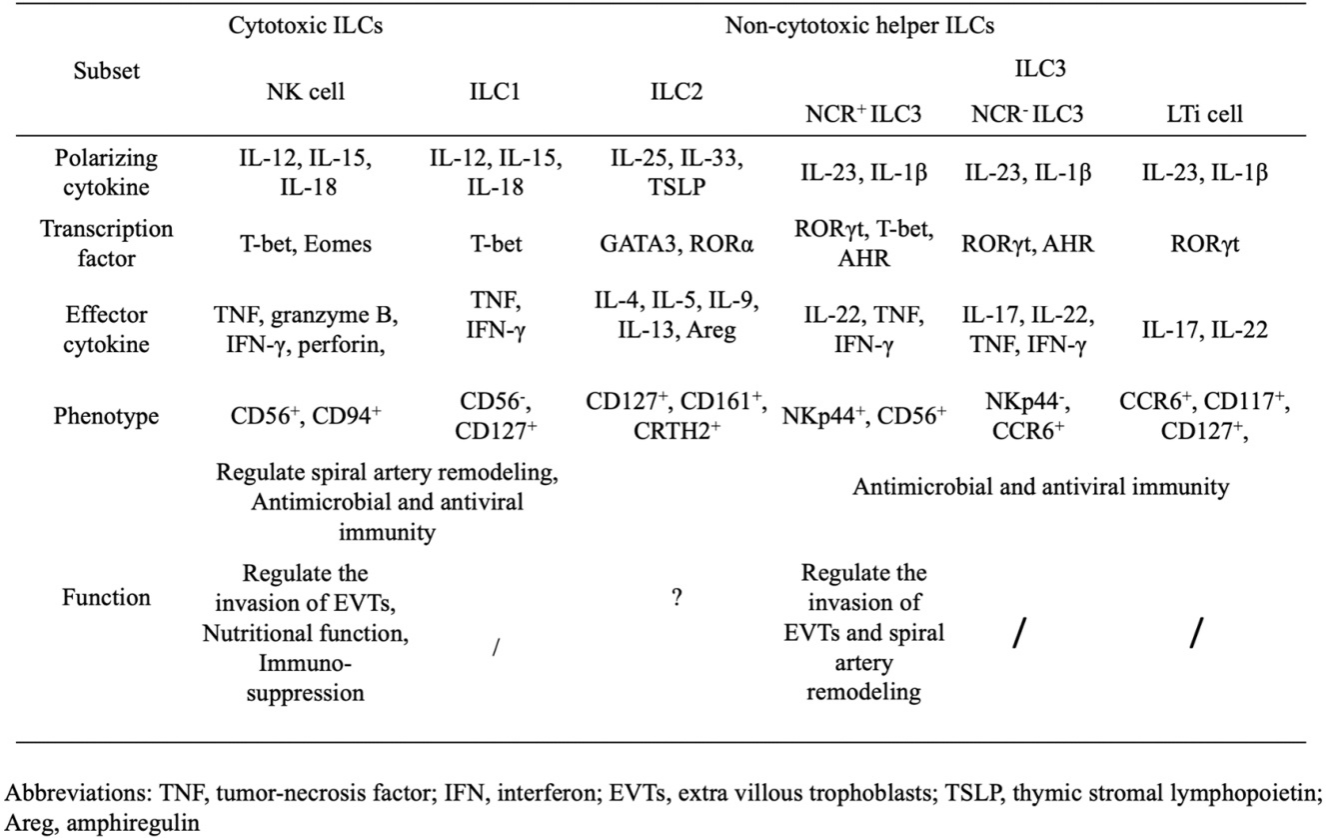

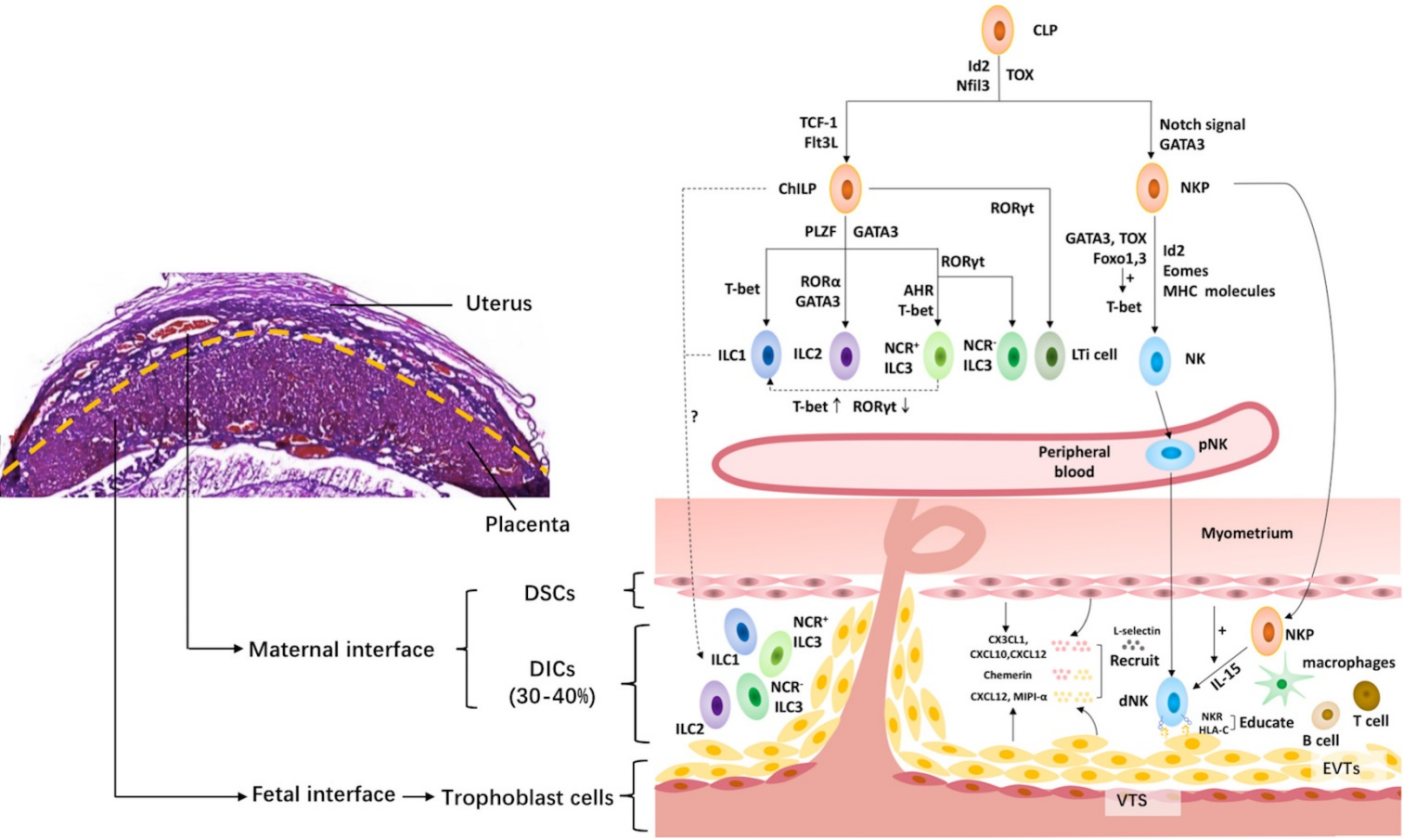
** The innate lymphoid cell family at the maternal-fetal interface.** The upper left shows the murine maternal-fetal interface stained by hematoxylin-eosin. And the right shows the innate lymphoid cell family at the maternal-fetal interface. All innate lymphoid cells (ILCs) arise from a common lymphoid progenitor (CLP). NK cells develop from a CLP via NK cell precursors (NKP), while the rest of the ILC subsets develop from a CLP via common helper ILC precursors (ChILP). PLZF^-^ ChILP is restricted to lymphoid tissue inducer (LTi) cells, while PLZF^+^ ChILP are capable of differentiating into all non-cytotoxic helper ILC subsets, except for LTi cells. T-bet, GATA3 and RORγt are required for the lineage maintenance of NK cells, as well as ILC1s, ILC2s and ILC3s. The maternal-fetal interface is the frontier of the direct contact between the embryo and mother. It is mainly composed of fetal trophoblast cells, maternal decidual stromal cells (DSCs) and decidual immune cells (DICs), including ILCs, macrophages, T cells, B cells, etc. At the maternal-fetal interface, all the ILC subsets are identified during the third trimester, while only NK cells, ILC1s and ILC3s are present in early pregnancy. The origin of dNK cells contains *in situ* progenitors, as well as peripherally derived HPCs and/or pNK homing cells. The migration of NK cells from the periphery to the decidua requires chemokines, including CXCL12 and MIPI-α, secreted by trophoblasts cells, and CX3CL1, CXCL10 and CXCL12 secreted by DSCs, adhesion molecules, such as L-selectin, as well as chemerin expressed in DSC and extravillous trophoblast cells. However, little is known about the origin of the rest of the ILC subsets in the human decidua. Moreover, NK cells acquire functional competence and self-tolerance by NK cell education via constant NK receptor (NKR)-MHC interactions. Id2, inhibitor of DNA binding 2; Flt3L, Flt3 ligand; GATA3, GATA-binding protein 3; PLZF, promyelocytic leukemia zincfinger protein; RORα, retinoic acid receptor-related orphan receptor-α; RORγt, retinoid-related orphan receptor γt; AHR, aryl hydrocarbon receptor; Eomes, Eomesodermin; NCR, natural cytotoxicity receptor; DSC, decidual stromal cells; NKR, natural killer receptors; VTS, villous trophoblasts; EVTs, extravillous trophoblasts.

The maternal-fetal interface is the frontier of the direct contact between the embryo and mother (Fig. 1). It is mainly composed of fetal trophoblast cells, maternal decidual stromal cells (DSCs) and decidual immune cells (DICs) 33.

### Fetal Trophoblast Cell

Human trophoblast cell, the main components of placenta, is divided into two main cell lineages, namely, villous trophoblasts (VTS) and extravillous trophoblasts (EVTs). VTS form chorionic villi, cover the surface of the villi which transports nutrients and oxygen to the fetus, and produce a variety of hormones and pregnancy factors that are required for the development and maintenance of embryos, such as human chorionic gonadotropin (HCG), progesterone and human placental lactogen, neurotransmitters, inhibin and activin. EVTs directly contact with the immune cells of the mother's decidua. They invade the decidua tissue, remodel the spiral artery and intrude into the blood vessels. The invasion of EVT breaks the contractility of spiral arteries for ensuring sufficient blood supply in the placenta 34. Therefore, the invasion of EVT is an essential process for fetal implantation and placenta formation.

### Maternal DSC

DSCs, the main constituent of the decidua, are differentiated from the fibroblast-like precursor cells of nonpregnant endometrium under the induction of estrogen and progesterone. In addition to the nutrient supply in decidua, DSCs also secrete hormones (e.g., prolactin), cytokines, and enzymes; expresses the progesterone receptor; and regulate embryo implantation and placental development. As potential immune cells, DSCs secrete a variety of cytokines and play an important role in immune regulation 35. By secreting CXCL12, DSCs promote the accumulation of peripheral NK cell in decidua and induce the conversion of pNK to dNK-phenotype 36-39. Besides, DSCs contribute to Th2 bias at maternal-fetal interface by producing CCL2 and IL-33. DSC-secreted CCL2 also participates in immunosuppression by inhibiting the cytotoxicity of NK cells during pregnancy 40.

### Maternal DIC

The composition of DICs is quite special. During early pregnancy, DICs account for 30-40% of the decidual cells. Among them, decidual NK (dNK) cells reach up to 70%, macrophages account for 20%, T cells account for 10%, and dendritic cells and B cells account for a smaller percentage. By interacting with each other and restricting each other, the DICs form a special immune network in the decidual microenvironment. In early pregnancy, to protect the semi-allogeneic fetal placenta from attacking by the maternal immune system, the main role of interactions between DICs is to maintain immune suppression; while, during late pregnancy, that transforms to immune rejection in order to prepare for fetal delivery. Therefore, the number and function of the DICs are changing in different stages of pregnancy 41. In a normal pregnancy, dynamic changes in the DICs-formed network are required to meet the physiological needs in different periods of pregnancy. Once the balance of the system is broken, it inevitably leads to serious consequences, such as abortion, premature delivery, intrauterine growth retardation and preeclampsia. Therefore, the balance of the DICs-formed network is crucial to the success of pregnancy 42.

## ILCs at the Maternal-Fetal Interface

It is well known that NK cells are the main components of the immune system at the maternal-fetal interface. In 1991, the presence of dNK cells was characterized during early placentation 43. With the increasing focus on the ILCs, other subsets were identified in the human decidua of early pregnancy (Fig. 1) 44-46.

### Decidual NK Cells

Similar to other lymphocytes, the ILC family arises from a common lymphoid progenitor (CLP). Notably, NK cells develop from a CLP via NK cell precursors (NKP), while the rest develop from a CLP via common helper ILC precursors (ChILP)^15^,^47^. Recently, strict NKP, which represent a separate downstream branch of CLPs and only generate cytotoxic NK cells, were defined as a Lin^-^CD34^+^CD38^+^CD123^-^CD45RA^+^CD7^+^CD10^+^CD127^-^ population and were identified in the human umbilical cord blood, bone marrow, fetal liver, and adult tonsil 48. Although the complex network that regulates the NK-specific differentiation of CLPs remains unclear, several transcription factors have been revealed. Studies in *Nfil3*-deficient mice observe a specific lack of NK cells, which demonstrate the necessity of *Nfil3* in NK cell lineage differentiation 49, 50, and *Nfil3* is required only when the CLP differentiation is toward NKP 51. Id2, a transcriptional suppressor of E protein activity, is essential for the generation of NK cells at the NKP stage 10, 11 and NK cell maturation 10, 52 in mice. Moreover, the differentiation from CLP to NK cells is promoted by Notch ligands Jagged1 in humans and Jagged2 in mice, respectively 53, 54. For NK cell maturation, studies in mice reveal that T-bet and some other regulatory factors play a central role in this transition. A deficiency of T-bet results in decreased expression of the transcription factor Blimp1 55, arrest at the immature stage, and a lack of mature NK cells in the bone marrow and periphery 56. Some other transcription factors, such as the positive regulator GATA3 57 and TOX 58, 59, as well as the suppressors Foxo1 and Foxo3 60, regulate the expression of T-bet and, thus, participate in NK cell maturation in humans. Eomes, another T-box transcription factor family member, is also crucial for NK cell terminal maturation, as murine mature NK cells revert to phenotypic immaturity with the deletion of Eomes 17. Meanwhile, NK cell maturation is also regulated by MHC molecules 61, which are a combination of polymorphic HLA-C as well as oligomorphic HLA-E and HLA-G molecules expressed by the placental trophoblast cells during human pregnancy 62. However, despite intense research, how tissue signals control the NK-specific differentiation of CLPs via transcriptional factors is still not fully understood.

During pregnancy, the number of dNK cells accessed peaks in the first trimester (over 70% of decidual leukocytes), then decreases gradually and reaches the bottom at term 43, 63. Since the concept that dNK cells represent a distinctive, hormonally regulated subset was proposed in 1991, numerous studies have investigated these cells for decades. NK cells in the human decidua are CD56^superbright^CD16^-^, and have a distinct phenotype compared to peripheral blood NK (pNK) cells. CD56^superbright^ dNK cells express CD9 and CD49a, while pNK cells are CD9^-^CD49a^-^ 8, 64-66. Except for the widely used classification according to the expression level of CD56, NK cells are also divided into four subsets according to the expression of CD11b and CD27: CD11b^+^CD27^-^ with high cytotoxicity; CD11b^-^CD27^+^ as well as CD11b^+^CD27^+^ with best ability to secret cytokines; and CD11b^-^CD27^-^ with differentiation potential 67-69. In humans, it is reported that more than 90% of pNK cells are CD11b^+^CD27^-^, while for dNK cells, approximately 60% are CD11b^-^CD27^-^ and over 20% are CD27^+^ 68. Moreover, natural killer group 2 (NKG2)A/C/E and killer-cell immunoglobulin-like receptors (KIR) show higher expression levels on dNK cells than those on pNK cells 65, 70, 71, while the T-cell immunoglobulin domain and mucin domain-containing molecule-3 (Tim-3) show lower expression levels on dNK cells than those on pNK cells 72.

As a distinct subset with unique phenotypic and functional features, the origin of dNK cells is not fully understood. One possibility is that dNK cells derive from recruited pNKs with the adaptation of the microenvironment in decidua. The conversion of the pNK to dNK-phenotype can be induced when pNK cells are cultured with DSCs or TGF-β1 73, 74. The migration of NK cells from the periphery to the decidua requires chemokines (CXCL12 39, 75 and MIPI-α 76, secreted by trophoblasts cells, and CX3CL1/fractalkine, CXCL10/IP-10 and CXCL12/SDF-1, secreted by DSCs 77), adhesion molecules (such as L-selectin 78), as well as extravillous trophoblast and DSC-derived chemerin 79. Additionally, the accumulation of NK cells in the decidua is promoted with the increasing level of estradiol (E2), luteinizing hormone and progesterone during pregnancy 80. Peripherally derived hematopoietic progenitor cells are another possible origin of dNK cells, considering the presence of donor-derived dNK cells in the murine uteri during decidualization following experimental transgenic labeling of transplanted bone marrow cells 81, 82. Based on the presence of CD34^+^ hematopoietic precursors with the potential to give rise to functional CD56^bright^CD16^-^ NK cells in the human decidua, the possibility that dNK cells arise from *in situ* progenitors is proposed 83. Additionally, tissue-specific differentiation of resident progenitors occurs in the presence of IL-15 84 or upon co-culture with DSCs 83. Thus, the origin of dNK cells is quite complex and is thought to contain *in situ* progenitors, as well as peripherally derived hematopoietic progenitor cells and/or pNK homing cells 66, 85, and needs more evidence to clarify.

### Decidual Non-Cytotoxic Helper ILCs

In addition to NK cells, non-cytotoxic helper ILC subsets are identified in the human decidua, and their phenotypes are quite similar to those previously described. In humans, decidual ILC1s produce IFN-γ, express T-bet, and lacks CD56, Eomes and RORγt. Decidual NCR^+^ILC3s express CD56, CD117, CD127 and RORγt, produce a high amount of IL-8 and IL-22 but are negative for IFN-γ. Additionally, decidual LTi-like cells are Lin^-^CD56^-^NCR^-^, express IL17A as well as tumor necrosis factor, and express RORγt 8, 44. In subsequent publications, the presence of LTi-like cells and NCR^+^ILC3s in the human decidua are confirmed 45, 86, 87. However, the existence of decidual ILC1s is controversial. In a study by Doisne's group, ILC1s were undetected in the human endometrium or decidua 45. Moreover, Simoni and colleagues failed to detect any ILC1 population in human tissues and proposed the possibility of contamination by other cells, including T cells, ILC3s, dendritic cells, hematopoietic stem cells and NK cells, based on the t-distribution stochastic neighbor embedding analysis from the mass cytometry data 88. However, their analysis approach and ILC purification methods were questioned 89, 90. Thus, more advanced experimental techniques are needed for the precise definition and accurate proof of the existence of ILC1s in the future. Unlike ILC1s and ILC3s, ILC2s are detected as the most abundant ILC subset in the human decidua at the third trimester instead of the first trimester 46. Taken together, the distribution of decidual ILC subsets changes with the progress of pregnancy, which needs further attention.

Although all groups of non-cytotoxic ILCs are identified in the uterus of pregnant mice, only ILC1 is present in decidua. Other groups, however, can only be found in myometrium and the myometrial lymphoid aggregate of pregnancy. Thus, ILC1 is the only subpopulation of non-cytotoxic ILCs to interact directly with trophoblast in mice. Murine uterine ILC1s express CD49a, lack DX5, CD127 and TRAIL, and share similar pattern of transcription factor with human decidual ILC1s (T-bet^+^Eomes^-^RORγt^-^). Moreover, unlike human, mice lack NCR^+^ ILC3s in uterus 45.

Non-cytotoxic helper ILCs develop from ChILP, which is defined as expressing of Id2, CD127, and integrin α_4_β_7_, as well as lacking common lineage markers and CD25 91. Knockout mice have revealed several transcription factors that are essential for non-cytotoxic helper ILC development and function. Transcription factors, such as Nfil3 92, 93, TCF-1^94^, Flt3 ligand 95, Id2 11, 96 and TOX are necessary for the early development of ChILP 97. In addition, the transcription factor promyelocytic leukemia zinc finger protein (PLZF) is essential for all non-cytotoxic helper ILC subsets, except for LTi cells, during their development 98, 99, and GATA3 is critical for the generation of PLZF^+^ ILC progenitors 100. T-bet, GATA3 and RORγt are the master transcription factors for the maturation of ILC1s 91, ILC2s 25 and LTi-like cells 32, 101, 102, respectively. T-bet, RORγt and AHR are among the factors that then control the final stages of NCR^+^ILC3 cell maturation 32, 102, 103. Notably, unlike NK cells, which never express RORγt during their development, there is a rapid increase in T-bet expression and a gradual decrease in RORγt expression during the development of a group of ILC1s 29, 104. Indeed, this ILC1 population expresses a high level of T-bet and a low level of RORγt 104. Taken together, the plasticity between ILC1s and ILC3s may exist, and complicate the definition of ILC1s.

Although there are several reports about the origin of NCR^+^ILC3s, little is known about the origin of non-cytotoxic helper ILCs in the human decidua. Marina *et al*. found that culturing peripheral or cord blood cells led to the failure of NCR^+^ILC3 production 27. In accordance with this, human RORγt^+^CD34^+^ cells, the lineage-specified progenitors of NCR^+^ ILC3s, are located in the tonsil and intestinal lamina propria but not the peripheral blood, thymus, or bone marrow 105, which indicates the possibility that tissue-specific NCR^+^ILC3s originate *in situ*.

## Functions of Decidual ILCs in Normal Pregnancy

**Figure 2:**
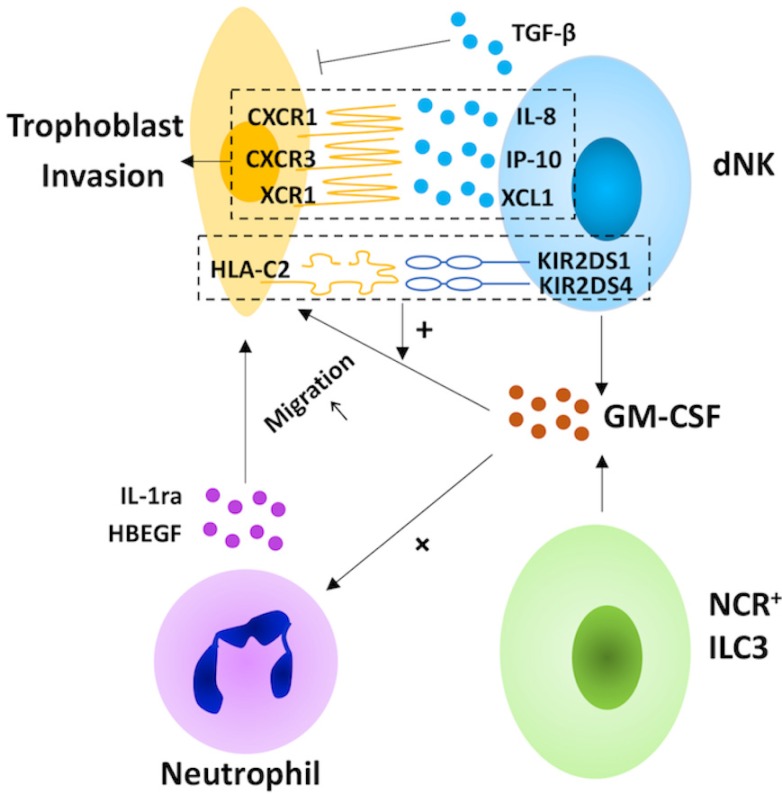
** The role of decidual innate lymphoid cells in trophoblast invasion.** In the human decidua, natural killer (NK) cells and group 3 innate lymphoid cells (ILC3s) participate in the invasion of extravillous trophoblast cells (EVT). Decidual NK (dNK) cells play a bi-directional regulatory role in trophoblast invasion. On the one hand, dNK cells direct this process by secreting chemokines (including interleukin (IL)-8, interferon-inducible protein (IP)-10 and XCL1) and granulocyte macrophage colony-stimulating factor (GM-CSF), which increase in response to the ligation of the NK cell-activating receptors KIR2DS1 and 4. On the other hand, NK cells also inhibit EVT invasion via TGF-β secretion. For NCR^+^ILC3s, the production of GM-CSF promotes trophoblast migration directly, as well as by inducing the expression of heparin-binding epidermal growth factor-like growth factor (HBEGF) and IL1ra in neutrophils.

**Figure 3:**
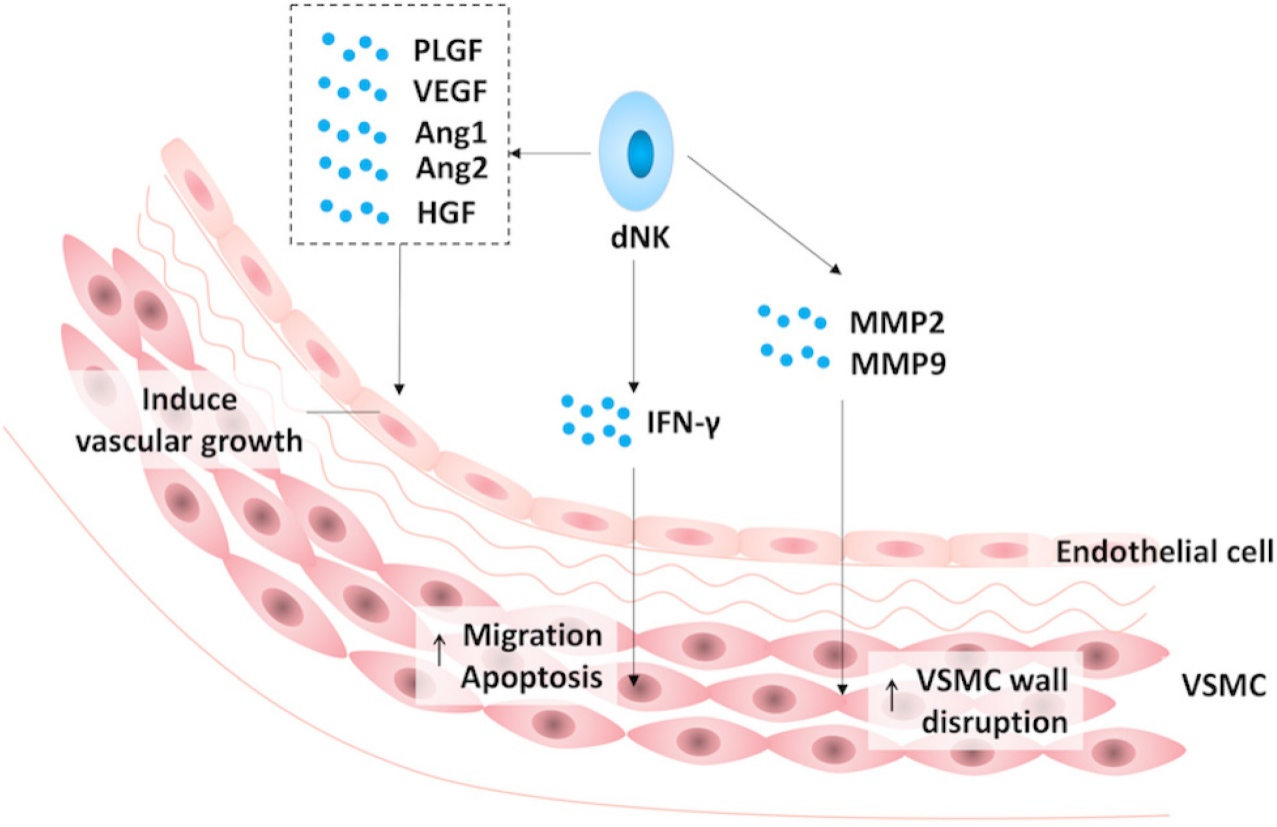
** The role of decidual natural killer cells in spiral artery remodeling.** In early pregnancy, decidual natural killer (NK) cells promote the process of spiral artery remodeling by inducing vascular growth via angiogenic factors, including vascular endothelial growth factor (VEGF), angiopoietin (Ang) 1 and 2, placental growth factor (PLGF) and hepatocyte growth factor (HGF), enhancing the migration and apoptosis of vascular smooth muscle cell (VSMC) layers via interferon-γ (IFN-γ) as well as facilitating the disruption of the VSMC wall via matrix metalloproteinase (MMP) 2 and 9.

As an arm of the innate immune system, ILCs play an important role in the suppression of fetal rejection, trophoblast invasion (Fig. 2) and spiral artery remodeling (Fig. 3) during pregnancy.

In humans, the invasion of EVTs into the maternal decidualized uterus is vital for the establishment of pregnancy 106. Decidual NK cells may direct this process by producing chemokines, including IL-8, the ligand for CXCR1 expressed on invasive trophoblasts, IP-10, one of the ligands of the CXCR3 expressed on trophoblast cells, and XCL1, the ligand for XCR1 expressed by EVT 107, 108. Moreover, dNK cell-derived granulocyte macrophage colony-stimulating factor (GM-CSF), which induces trophoblast migration, increases in response to the ligation of the NK cell-activating receptors KIR2DS1 and 4 108, 109. The facilitation of dNK cells in trophoblast invasion is also promoted by the cross-talk between dNK cells and invasive trophoblast cells, such as the binding of maternal KIR2DS1 and fetal HLA-C2 109, 33. Paradoxically, dNK cells inhibit EVT invasion via TGF-β secretion 110. Taken together, dNK cells may play a bi-directional regulatory role in trophoblast invasion (Table 1). Except for dNK cells, trophoblast invasion is also regulated by NCR^+^ILC3s in the human decidua. NCR^+^ILC3-derived GM-CSF promotes trophoblast migration directly, as well as by inducing the expression of heparin-binding epidermal growth factor-like growth factor (HBEGF) and IL1ra in neutrophils 108, 111-113. Thus far, whether decidual LTi-like cells are capable of regulating trophoblast migration is still unknown. However, it is reported that IL-17 promotes trophoblast proliferation and invasion 114. In light of this, decidual LTi-like cells may promote trophoblast invasion by secreting IL-17, and this warrants further investigation.

Adequate perfusion is the cornerstone of pregnancy maintenance as well as fetal growth. It requires spiral artery remodeling, which is partly regulated by decidual ILCs. Evidence from murine models indicates that the initiation of spiral artery remodeling requires IFN-γ, which is one of the representative cytokines produced by NK cells and ILC1s. Histological observations reveal that IFN-γ promotes the growth of the decidual vascular lumen size and facilitates the separation of vascular smooth muscle cell (VSMC) layers 115, 116. Quite recently, it was demonstrated that IFN-γ promotes the migration and apoptosis of VSMCs during vascular transformation 117. In addition to IFN-γ, dNK-derived matrix metalloproteinases (MMP) 2 and 9 also mediate the disruption of the VSMC wall during pregnancy 118. Meanwhile, human dNK cells are capable of secreting several angiogenic factors, which induce vascular growth and participate in spiral artery remodeling, including vascular endothelial growth factor (VEGF), angiopoietin 1 and 2, placental growth factor and hepatocyte growth factor 107, 119, 120. In addition, decidual NCR^+^ILC3s are involved in angiogenesis by secreting cytokines, such as GM-CSF and CXCL8, to upregulate the expression of HBEGF and IL1ra in neutrophils 111-113.

During early pregnancy, the nutritional function of the placenta is not yet perfect. CD49a^+^Eomes^+^ NK cells, the dominant population of dNK cells, secrete growth-promoting factors, such as pleiotrophin and osteoglycin, and participate in optimizing the utilization of maternal nutrition in order to meet the needs of fetal growth 8. Moreover, dNK cells promote trophoblast growth and differentiation by secreting cytokines, including IL-22 121, GM-CSF and colony-stimulating factor-1 in mice 122-124.

During pregnancy, the survival of semi-allogeneic fetal tissue requires a sophisticated immune regulatory network. It is commonly believed that specialized regulation may occur depending on specific anatomic site during pregnancy. By inducing the generation and differentiation of regulatory T cells via TGF-β, as well as inhibiting Th17-mediated local inflammation via IFN-γ, dNK cells promote maternal tolerance against the semi-allogeneic fetus 119, 125-129. IL-22 is a key regulator in immunity, inflammation and tissue homeostasis, and is closely related to the occurrence and development of several autoimmune diseases, including inflammatory bowel disease, psoriasis and graft versus host disease, as shown in patient or animal models 130-136. In view of this, it is possible that decidual NCR^+^ILC3s exert suppression on fetal rejection by secreting IL-22, but so far direct evidence for this is lacking.

On account of the increased susceptibility and severity to some infections (Among pregnant women, the role of decidual ILCs in defense against infection is quite important 137. NK- or ILC1-derived IFN-γ, for example, is crucial in antimicrobial and antiviral immunity by activating macrophages, enhancing antigen presentation and promoting Th1 differentiation 138. Moreover, ILC3s are also an important source of GM-CSF, which recruits inflammatory monocytes and acts to prevent infection. In a mouse model of intestinal inflammation, ILC3 depletion partially impairs the ability to control bacterial infection 139. Further study is required to determine whether ILC3s have the same anti-infection effect at the maternal-fetal interface.

## Regulation of Decidual ILC Differentiation

**Figure 4:**
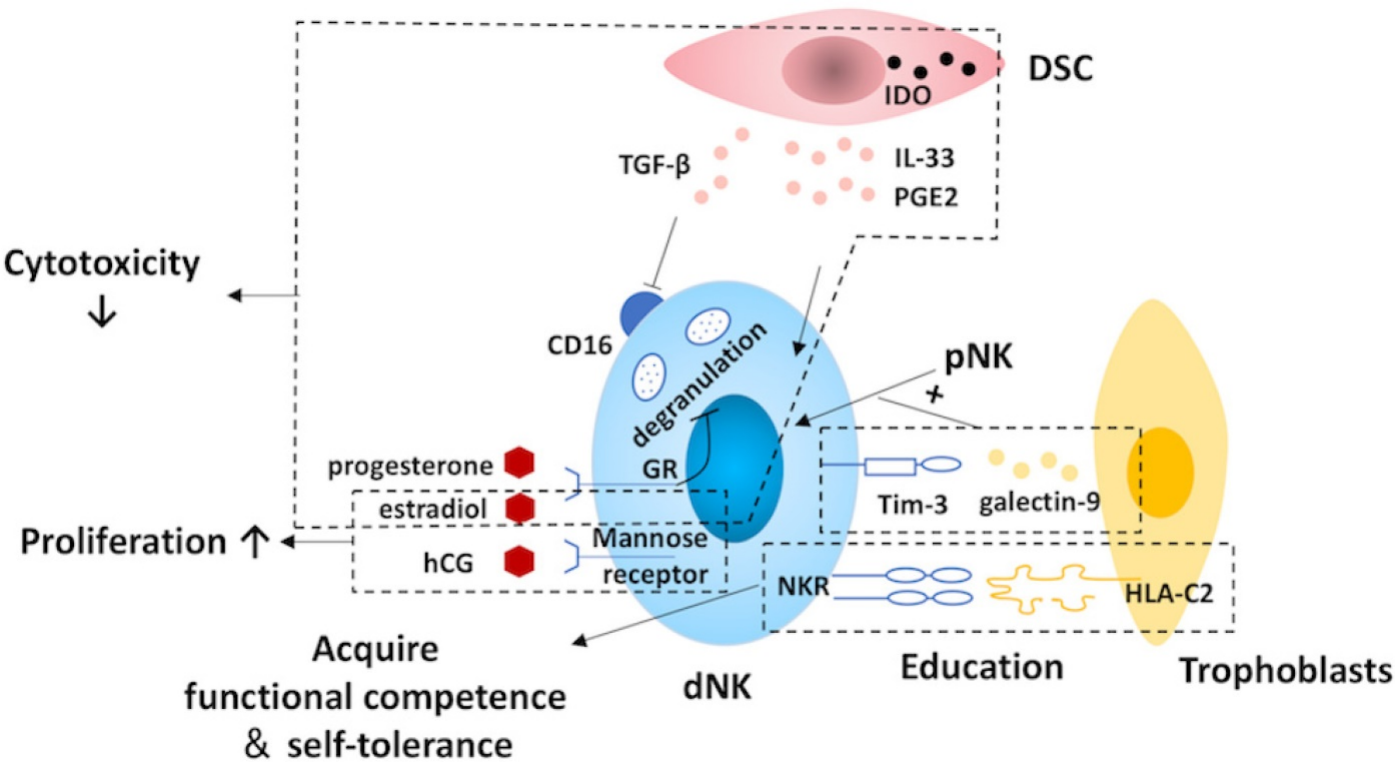
** Regulation of decidual natural killer cells.** At the maternal-fetal interface, natural killer (NK) cells are regulated by trophoblasts, decidual stromal cells (DSCs) and hormones. NK cells acquire functional competence and self-tolerance by NK cell education via the constant interactions between NK receptors (NKR) and the MHC molecules expressed by the trophoblast cells. Moreover, trophoblasts also promote the conversion of recruited pNK to dNK cells via the galectin-9/Tim-3 pathway. The expression of CD16 on NK cells is suppressed by DSC-derived TGFβ. Additionally, DSCs are capable of inhibiting the cytotoxicity of NK cells by expressing indoleamine 2,3-dioxygenase (IDO) as well as secreting IL-33 and prostaglandin E_2_ (PGE_2_). Also, hormones and pregnancy factors produced by trophoblasts, such as human chorionic gonadotropin (hCG), progesterone and estradiol, play a role in dNK regulation. The cytotoxicity of NK cells is suppressed by progesterone via blocking degranulation and estradiol. In addition, the proliferation of dNK cells is promoted by hCG and estradiol. Tim-3, T-cell immunoglobulin domain and mucin domain-containing molecule-3; dNK, decidual natural killer; pNK, peripheral blood natural killer.

The maternal-fetal interface is the core of the mother-fetal dialogic regulatory network, where trophoblast cells, DSCs, DICs, and various cytokines and hormones produced by these cells, with interaction dialogues, jointly construct the special immune tolerance microenvironment, in order to maintain physiological pregnancy. Therefore, trophoblast cells, DSCs and hormones in the microenvironment at the maternal-fetal interface are bound to regulate the differentiation and function of the decidua ILCs. However, research on these regulatory mechanisms is mainly focused on dNK cells (Fig. 4), and little is known about the regulation of non-cytotoxic helper ILC differentiation in the human decidua.

The fetal trophoblasts actively educate the dNK cells. Through constant NK receptor-MHC interactions, NK cells acquire functional competence and self-tolerance, and this process is defined as NK cell education 140, 141. Herein, the education is much more sophisticated at the maternal-fetal interface both in humans and in mice, as it is an important physiological situation in which allogeneic (paternal) MHC class I molecules are presented 66, 142. Human genetic epidemiological data and mouse studies suggest that the interactions between the NK receptors and the maternal environment, as well as the paternal MHC molecules expressed by the trophoblast cells regulate NK cell activity and somehow determine pregnancy outcomes 2, 142. In addition to NK cell education, trophoblasts also promote the conversion of recruited pNK to dNK cells via the galectin-9/Tim-3 pathway at the maternal-fetal interface during early pregnancy 72.

The DSC is another important regulator of dNK differentiation. CD16 is a surface marker that is tightly related to the cytotoxicity of NK cells 143, and its expression on pNK cells is down-regulated by DSC-derived TGF-β *in vitro* 73. By secreting IL-33, DSCs suppress the cytotoxicity of NK cells and induce the production of Th2 cytokines, including IL-4, IL-10, and IL-13 144. Additionally, DSC can inhibit the cytolytic activity of NK cells by expressing indoleamine 2,3-dioxygenase (IDO) and secreting prostaglandin E_2_ 145.

During pregnancy, the role of hormones cannot be neglected. Although, dNK cells lack steroid receptors and the classic luteinizing hormone/CG receptor, hormones can still exert their effects through the glucocorticoid receptor or the mannose receptor 146, 147. Evidence from experiments in mice suggests that E2 not only is favorable for NK cell proliferation but also can reduce their cytotoxicity 80, 148. Mediated by progesterone induced blocking factor, progesterone contributes to inhibition of dNK cell cytotoxicity via blocking degranulation 149, 150. Meanwhile, HCG also promotes the proliferation of dNK cells 147.

## Role of Decidual ILCs in Pathological Pregnancy

### Reproductive Failure

As the only subset of ILCs with cytotoxicity, dNK cells are lowly cytotoxic in adaptation to alloantigen stimulation during normal pregnancy 66, 151. Dysregulation of cytotoxicity can turn dNK cells into detrimental cells and cause reproductive failure, including spontaneous abortion, unexplained infertility and implantation failure after *in vitro* fertilization 152-154. The mechanism behind this connection is wildly discussed. For example, an imbalance between activating and inhibitory receptors on NK cells, specifically, the increased expression of NKG2D, an activating receptor that mediates NK cell cytotoxicity, as well as the lack of KIR, are deciding factors in pregnancy outcomes 155-157. Furthermore, NK cell impairment to suppress the expansion and activity of Th17 cells, is identified in patients with recurrent spontaneous abortion 129. Using the lipopolysaccharide-induced mouse abortion model, researchers found that in the uterus, a reduction of a tumor necrosis factor-like weak inducer of apoptosis (TWEAK) in dNK cells and the upregulation of its receptor, may alter the cytotoxicity of dNK cells and, ultimately, result in pregnancy loss 158. Quite recently, over-activated dNK cells, with an increased expression of angiogenic cytokines, such as VEGF-A and basic fibroblast growth factor, were observed in women with recurrent miscarriage, which suggests the potential relationship between the dysfunction of dNK cells in regulating angiogenesis and recurrent miscarriage 159.

The relationship between NCR^+^ILC3s and reproductive failure was also investigated. Kamoi *et al*. found that the percentage of decidual NCR^+^ILC3s in patients with unexplained recurrent spontaneous abortion was significantly higher than that in those with unexplained infertility 87. However, the lack of normal pregnant controls in this study requires further investigations to explore the exact relationship between NCR^+^ILC3s and reproductive failure.

### Preeclampsia

Preeclampsia, a serious pregnancy associated disease, is characterized by the insufficient activation of maternal NK cells as well as the excessive activation of decidual LTi-like cells during pregnancy 86, 160, 161. Many efforts have been made to explain the mechanism of preeclampsia. Several studies suggest that preeclampsia is a result of incomplete trophoblast invasion and spiral artery remodeling 162. While, some researchers believe that preeclampsia is related to a failure of the maternal vascular system to adapt the necessary volume load that occurs during pregnancy 163, 164. The ligation of KIR and HLA-C is a pivotal regulator for NK cell activation. A gene linkage analysis shows that the lack of activating KIR (AA genotype) on maternal NK cells, which in turn causes a strong inhibition of NK cells, is a risk factor for preeclampsia 160. Consistent with this, an additional MHC allele, which prefers binding to inhibitory rather than activating NK cell receptors, causes the suppression of NK cell activity and the incompetence of spiral artery remodeling in mice 142. Moreover, NCRs, including NKp44 and NKp46, may be potential predictive markers for preeclampsia, in view of the declined expression level of NCRs on pNK cells in women with preeclampsia, which is observed as early as 3-4 months before the onset of preeclampsia 165, 166. In addition, decidual LTi-like cells are shown as the main source of the increased IL-17 and lead to a higher risk of preeclampsia 86.

### Morbidly Adherent Placentation

Morbidly adherent placentation, such as placenta accreta, percreta and increta, is a life-threatening condition. It is reported that the amount of decidual natural killer (dNK) cells in patients with morbidly adherent placentation was significantly lower than normal pregnancy. Moreover, patients with adherent placenta previa showed lower dNK density compare with non-adherent placenta previa 167. This suggests that the deficiency of NK cells may participate in the process of morbidly adherent placentation. At the maternal-fetal interface, dNK cells inhibit the migration and column formation of extra villous trophoblasts by regulating protease activity and E-cadherin expression 168. Moreover, this inhibition can be reversed by anti-IFN-γ 168, 169. Thus, decidual NK cells might protect from morbidly adherent placentation by limiting the over invasion of the extra villous trophoblasts into the uterine wall. However, the relationship between non-cytotoxic helper ILCs and morbidly adherent placentation still remains a mystery and needs further research.

### Other Pregnancy-Associated Diseases

In addition to participating in preeclampsia, the ligation of KIR and HLA-C, as the essential regulator for NK cell activation, also affects fetal growth. Pregnancies are at an increased risk of intrauterine growth retardation not only in women with the KIR AA genotype but also in women with the presence of paternal H-2D^d^ 170. Additionally, it is reported that decidual LTi-like cells may participate in the development of gestational diabetes and chronic diabetes via secreting IL-17 86. Recently, the effect of IL-22 in the prevention of lipopolysaccharide-induced preterm labor in mice was revealed and, thus, presents a potential protective role of decidual NCR^+^ILC3s in inflammation-induced preterm birth 171. However, decidual ILC2s may play the opposite role in preterm birth due to the increase in ILC2s in the decidua basalis with spontaneous preterm labor compared to non-labor controls 46.

## Concluding Remarks

With a deeper understanding, ILCs in the human decidua are identified and are emerging as indispensable factors in the maintenance of normal pregnancy as well as the development of a wide range of pathological pregnancies (Table 1). Although dNK cells have been studied for decades, research regarding the non-cytotoxic helper ILCs present in the human decidual is still in its infancy. The relatively low number of non-cytotoxic helper ILCs present in the human decidua and the difficulty in the identification, separation and purification of the individual subsets may pose obstacles in further studies. However, given the great deal of similarities between ILCs and T cells in function, especially in the cytokine profile, existing studies from decidual T cells may contribute to shedding light on the exact role of decidual ILCs in human pregnancy.

## Figures and Tables

**Table 1:** Phenotypical heterogeneity and function of human ILCs.
